# Weighted Analysis of Corrosion-Inducing Impurities in Supercritical CO_2_ Transportation System

**DOI:** 10.3390/molecules31203631

**Published:** 2026-10-12

**Authors:** Chao Zhang, Tong Gao

**Affiliations:** CNOOC Petrochemical Engineering Co., Ltd., Qingdao 266000, China

**Keywords:** corrosion, impurities, meta-analysis, supercritical CO_2_

## Abstract

The degradation of carbon steels in supercritical carbon dioxide is significantly influenced by impurities, yet a comprehensive understanding of the corrosion mechanism and unified industry standards remains lacking. In response to this challenge, an impact factor weight analysis of 27 published articles, comprising 149 observations, was performed to evaluate the response of the general corrosion rate of carbon steels to impurities such as oxygen, sulfur dioxide, hydrogen sulfide, and nitrogen oxides, as well as their mixtures. The findings indicate that the impurities have distinct effects on steel degradation in concentration- and exposure-time-dependent manners. Notably, sulfur dioxide and nitrogen oxides demonstrate a significant impact, oxygen plays a moderate role, while hydrogen sulfide exerts a profound influence on the corrosion rate of carbon steels. Hydrogen sulfide and oxygen exhibit a synergistic effect that accelerates degradation in supercritical carbon dioxide, whereas no pronounced synergistic effect is observed for sulfur dioxide and oxygen. This study provides a quantitative ranking of impurity impacts and demonstrates the impact level of the synergistic effects of oxygen, sulfur dioxide, and hydrogen sulfide, offering a scientific basis for developing pipeline integrity management strategies in supercritical carbon dioxide environments.

## 1. Introduction

### 1.1. Background

Industrial and residential CO_2_ emissions have turned global warming into a major concern [1,2]. As the primary solution to eliminate CO_2_ emissions, carbon capture and storage (CCS) technology is a critical option. A general CCS process involves three stages: capture CO_2_ from industrial facilities, transport CO_2_ to storage sites, and inject CO_2_ into reservoirs [3]. In order to enhance transportation efficiency, CO_2_ is usually pressed into a supercritical state and transported in dedicated extensive pipelines [3,4,5].

One of the challenges of the transportation of supercritical CO_2_ is the internal corrosion of carbon steel pipelines. The corrosive nature of the supercritical fluid exacerbates the severity of corrosion of carbon steels and affects the design, fabrication, and maintenance of pipelines, posing a potential hazard during transportation [3,6].

### 1.2. Corrosion Mechanism

Established experience and laboratory studies indicate that water concentration plays a crucial role in steel corrosion. Notably, corrosion issues are rarely observed in pipelines where supercritical CO_2_ is thoroughly dehydrated, as evidenced by practices in enhanced oil recovery (EOR) transportation and monoethanolamine (MEA) extraction in the United States [1,7,8,9].

In contrast, in a supercritical CO_2_ environment where water is not removed, the corrosion mechanism is represented by the formation of FeCO_3,_ as shown in the following equation [3]:

(1) $$Fe+H_{2}O+CO_{2}\to Fe{CO}_{3}+2H_{2}$$

The specific mechanism is expressed as an electrochemical process that begins with the formation of an aqueous phase. The CO_2_-saturated water undergoes hydration to produce carbonic acid (H_2_CO_3_), which is a diprotic acid that dissociates according to the following reactions [1,10]:

(2) $$H_{2}CO_{3}\to H^{+}+{HCO_{3}}^{-},$$

(3) $${HCO_{3}}^{-}\to H^{+}+{CO_{3}}^{2-}$$

The primary cathodic reaction contributing to corrosion derives from the evolution of hydrogen, with three pathways given [1,10]:

(4) $$H^{+}+2e^{-}\to H_{2}\;\mathrm{or},$$

(5) $${2HCO_{3}}^{-}+2e^{-}\to H_{2}+2{CO_{3}}^{2-}\mathrm{or},$$

(6) $$H_{2}CO_{3}+2e^{-}\to H_{2}+{2HCO_{3}}^{-}$$

In a supercritical CO_2_ fluid, the free aqueous phase is naturally saturated with CO_2_ and exhibits a low pH value. Consequently, the cathodic reaction is extensively accelerated.

The anodic dissolution of iron is described in the following equation [11]:

(7) $$Fe\to Fe^{2+}+2e^{-}$$

Thus, in a supercritical CO_2_ environment where water is not completely removed, FeCO_3_ is generated. A dense layer of FeCO_3_ presents an effective protection to the substrate metal [12]. Nevertheless, this integrity is typically compromised in the presence of impurities within the bulk CO_2_.

### 1.3. The Influence of Impurities on the Corrosion of Carbon Steels

Impurities in water-saturated or under-saturated supercritical CO_2_ generally promote the degradation of carbon steels, as most impurities are associated with corrosive properties [13,14]. However, under specific circumstances, impurities may have an inhibitory effect on the corrosion rate. For instance, Hua et al. observed that O_2_ possessed a contrasting influence on degradation depending on the concentration of O_2_ in the bulk phase of supercritical CO_2_ [15,16]. Sun et al. [17] conducted a comprehensive investigation illustrating fluctuation in the corrosion rate over various diverse corrosive conditions. These studies indicate the intricate nature of corrosion mechanisms, as well as the challenges inherent in the attempt to interpret outcomes through a single experiment, as conclusions from a single experiment are constrained by material variability, environmental conditions, and testing methodologies.

The diversity of studies makes it difficult to draw a unified conclusion regarding the extent of influence on corrosion [1,18,19,20]. Additionally, the interactions among impurities exhibit complex synthetic effects. A universal criterion for impurity limit in supercritical CO_2_ transportation has not been well established in the CCS industry [18,21,22]. Consequently, a thorough understanding of the corrosion mechanism and the extent of the influence of impurities is required for the development of corrosion control strategies and the management of pipeline integrity.

Unlike individual case studies, meta-analysis systematically synthesizes quantitative findings from numerous independent research studies while addressing sources of heterogeneity and bias. Meta-analysis has been widely utilized for evaluating biological and ecological responses to changes [23]. However, such an approach has not been applied to assess the responses of corrosion to environmental changes due to the stringent demands for experimental protocols and sample replicates. Nonetheless, this methodological approach provides insights for the quantitative characterization of physical mechanisms based on extensive datasets.

Given the important alignment between methodological characteristics of meta-analysis and the diversity of operating conditions in engineering practices, this study used a meta-analysis approach to quantitatively evaluate the impact of impurities on carbon steel corrosion in water-saturated or under-saturated supercritical CO_2_ environments while elucidating corrosion mechanisms. To the best of our knowledge, this is the first application of meta-analysis to systematically quantify the factors influencing corrosion in this field. Subgroup analysis and heterogeneity assessment were adopted to identify the key influencing factors from the dispersed and extensive datasets.

Corrosion impact factor (CiF), which is defined as the difference in the corrosion rate in the target environment and the standard reference environment divided by the corrosion rate at the standard reference, is applied to illustrate the extent of influence of the impact factors. The general corrosion rate was selected as the response variable to reflect the extent of degradation of steels. The responses of the corrosion rate to the impurities were extensively reported in the past two decades. Compared with localized or pitting corrosion rate, the general corrosion rate was considered dependable since its measurement derived from a reproducible and standardized method that followed well-established protocols [18].

A database comprising 27 publications with 149 studies was utilized to investigate the effects of impurities on the corrosion rate. The following hypotheses were proposed:

(1) The differences in the corrosion rate among samples within the same comparison reflected the influence of experimental conditions imposed on steels.

(2) Under limited conditions where supercritical CO_2_ was either water-saturated or under-saturated at ambient temperature, additional influence factors such as pressure and temperature did not bring about detrimental changes to the observed patterns of influence.

## 2. Results

Supplementary Table S2 presents the overall impact of impurities on the corrosion rates in water-saturated and under-saturated supercritical CO_2_ (categorized by concentration). Supplementary Table S3 presents the overall impact of impurities on the corrosion rates in water-saturated and under-saturated supercritical CO_2_ (categorized by exposure time). Supplementary Table S4 presents the overall impact factor and 95% confidence intervals for different CV values of the influence of impurities on corrosion in supercritical CO_2_ (categorized by concentration). Supplementary Table S4 presents the overall impact factor and 95% confidence intervals for different CV values regarding the influence of impurities on corrosion in supercritical CO_2_ (categorized by exposure time).

For Figure 1a,b, Figure 2a,b, Figure 3a,b, Figure 4a,b, Figure 5a,b, Figure 6a,b and Figure 7a,b, each symbol represents the mean response ratio, while the error bars represent the 95% confidence intervals (CIs). The sample size for each variable is displayed in parentheses next to each bar. The mean bar values greater than zero suggest a positive effect on the general corrosion rate. Figure 1a,b shows the overall effect sizes for the four impurities (O_2_, SO_2_, H_2_S and NO_x_) across subgroups categorized by concentration and exposure time.

### 2.1. The Overall Influence of O_2_ on the Corrosion Rate in Supercritical CO_2_

The introduction of O_2_ slightly increased the corrosion rate of carbon steels (Figure 2a). An overall corrosion impact factor of 0.8729 demonstrated a positive correlation between the presence of O_2_ and the corrosion rate. Across the entire dataset, O_2_ increased the corrosion rate of carbon steels in a concentration-related manner. The *p* value between groups of 0.0116 (<0.05) indicated that CiF differed among subgroups with different concentrations: higher responses to the corrosion rates were observed in comparisons with O_2_ concentrations above 0.1% (2.2343). In contrast, no significant change in the corrosion rate was observed when O_2_ concentrations were between 0.05% and 0.1% (0.2193) and O_2_ concentration was below 0.05% (0.6199).

The influence of O_2_ on the corrosion rate was analyzed in response to different exposure times (Figure 2b). Across the 39 included studies, the pooled CiF indicated a notable divergence (*p* between groups = 0.0004). Comparisons involving longer exposure times (≥72 h) demonstrated a stronger response with a CiF of 2.0536, suggesting that the pipeline immersed in O_2_-contaminated supercritical CO_2_ experienced long-term and sustained influence. However, a contrasting trend of response to the exposure time was observed in the subgroup with a duration of less than 24 h (0.7575) and the subgroup with a duration of 24 to 72 h (−0.1377). Specifically, with the introduction of O_2_ to supercritical CO_2_, the general corrosion rate almost doubled in the initial 24 h, but this effect diminished over the subsequent 48 h.

### 2.2. The Overall Influence of SO_2_ on the Corrosion Rate in Supercritical CO_2_

A notable response to the presence of SO_2_ in supercritical CO_2_ was observed in the corrosion rates of carbon steel across subgroups with varying SO_2_ concentrations (Figure 3a). A higher CiF was observed in subgroups when the concentration of SO_2_ was above 0.1% (44.4151) and between 0.05% and 0.1% (14.8982). When the concentration of SO_2_ was below 0.05%, a CiF of 6.5830 was observed through the analysis. Despite differences in the effect size, the *p* between groups was 0.1730 (>0.05), indicating that unexplained variability persisted among individual studies within each concentration subgroup.

Twenty-five observations examining the influence of SO_2_ in response to various exposure times revealed that the influence of SO_2_ declined with exposure duration (Figure 3b). The introduction of SO_2_ significantly increased the general corrosion rate by 44.9161, 9.9752, and 8.0304, respectively, with prolonged exposure time. Additionally, the *p* value between groups of 0.2184 (>0.05) indicated unaccountable variables remained among individual studies within subgroups.

### 2.3. The Overall Influence of H_2_S on the Corrosion Rate in Supercritical CO_2_

A higher CiF was observed in the subgroups with H_2_S concentrations below 0.1% (2.2008 and 2.7279) than in the subgroup with H_2_S concentration above 0.1% (0.6575) (Figure 4a). The data analysis indicated a decrease in the corrosion rate when the H_2_S concentration exceeded a specific threshold. Nevertheless, from the perspective of heterogeneity, considerable within-subgroup heterogeneity persisted (*p* between groups = 0.5954).

H_2_S exerted an accelerating effect on the corrosion rate of carbon steels in the supercritical CO_2_ environment, and this effect intensified with prolonged exposure (Figure 4b). Across the 22 comparisons, the impact significantly increased when the exposure time exceeded 72 h (2.9785); in contrast, when the exposure time ranged from 24 to 72 h, the CiF was −0.0711. A *p* value between groups of 0.0810 indicated a moderate difference between the subgroups.

### 2.4. The Overall Influence of NO_x_ on the Corrosion Rate in Supercritical CO_2_

Significant positive correlations were detected between the presence of NO_x_ and the corrosion rate (Figure 5a). The responses of the corrosion rate varied among the subgroups with different concentration ranges. A pronounced degradation of carbon steels was observed when the concentration of NO_x_ was below 0.05% (14.4485). The effect size was 21.9753 when the concentration of NO_x_ ranged from 0.05% to 0.1%. The response of the corrosion rate to NO_x_ at a concentration above 0.1% was not evaluated due to insufficient experimental data. The *p* value between the two subgroups was 0.7390 with a high within-group value of 23.7425, suggesting significant heterogeneity within subgroups.

The influence of NO_x_ on the degradation of carbon steels in response to various exposure times was evaluated through analysis (Figure 5b); however, the insufficient number of observations in each subgroup limited the statistical power of the analysis.

In terms of effect size, the influence of NO_x_ intensified with exposure duration, with moderate differences observed between the subgroups, and the *p* value between the two subgroups was 0.1326.

### 2.5. Synergistic and Interactive Influence of O_2_ and SO_2_ on the Corrosion Rate in Supercritical CO_2_

The effect of SO_2_ was evaluated within subgroups of the CO_2_-O_2_ and CO_2_ systems. The *p* value between subgroups of 0.8666 (>0.05) implied no distinction between the two subgroups (Figure 6a), with statistically similar corrosion impact factors of 11.3968 and 12.2711, respectively.

The effect of O_2_ on the corrosion rate in the supercritical CO_2_ was investigated when SO_2_ was presented and SO_2_ was absent (Figure 6b). The CiF with only O_2_ closely resembled the overall CiF of O_2_ for corrosion rates (1.0850), whereas the subgroup with SO_2_ exhibited an apparent decrease (0.2931). The *p* between-groups value of 0.1024 suggested a slight distinction between the two subgroups.

### 2.6. Synergistic and Interactive Influence of O_2_ and H_2_S on the Corrosion Rate in Supercritical CO_2_

The effect of H_2_S on the general corrosion rate was analyzed in the O_2_-CO_2_ system and the CO_2_ system (Figure 7a). No significant difference was detected between the two subgroups (the *p* value between groups = 0.2680) regarding the presence of O_2_. A diminished response was observed in the absence of O_2_ (1.6801) compared to its presence (3.5475). The former value was close to the overall CiF of H_2_S for the corrosion rate (1.9185).

The corrosion impact factors of O_2_ on the general corrosion rate of two subgroups (with and without H_2_S) were 1.8470 and 0.7367, respectively (Figure 7b). Compared with O_2_, H_2_S exerted a more substantial impact on corrosion. In the subgroup analysis, the CiF in the H_2_S-absent subgroup was close to the overall response observed in O_2_.

## 3. Discussion

It is generally believed that for carbon steels submerged in supercritical CO_2_, the dense and adherent surface of FeCO_3_ can be compromised or partially transformed into a porous, flaky, or rusty product following the introduction of impurities. O_2_, SO_2_, H_2_S, and NO_x_ alter the corrosion mechanism of metals and affect the degree of degradation. Consequently, a systematic and accurate quantitative evaluation is constructed to determine the extent to which each impurity contributes to corrosion and gain insights into the corrosion mechanism.

### 3.1. The Comprehensive Impact of O_2_ on the Corrosion Rate in Supercritical CO_2_

O_2_ is derived from the air as a general component of combustion in CCS practices. O_2_ facilitates corrosion through two primary mechanisms: Firstly, the presence of O_2_ introduces an additional cathodic reaction, which increases the relative humidity of the bulk phase due to the formation of H_2_O. Secondly, O_2_ triggers the creation of iron oxides and hinders the formation of protective film by forming porous iron oxides, leaving more surface unprotected [17,24,25].

(8) $$O_{2}+4H^{+}+4e^{-}\to H_{2}O,$$

(9) $${2Fe}^{3+}+3{OH}^{-}\to {Fe}_{2}O_{3}+3H^{+}$$

To further validate the hypothesis, most studies employed scanning electron microscopy (SEM) to analyze morphology, with or without energy-dispersive spectroscopy (EDS), to analyze the elemental composition of the corrosion products. Wang et al. reported that carbon steel samples submerged in supercritical CO_2_ without O_2_ exhibited a uniform film on the surface, with the atomic ratio corresponding to FeCO_3_, while samples that were submerged in supercritical CO_2_ with O_2_ exhibited worm-like products, with distinctly higher Fe ratio [26]. Sun et al. confirmed that the worm-like products contained Fe_2_O_3_ [27].

A notable increase in the corrosion rate responses was detected at O_2_ concentrations exceeding 0.1% through the meta-analysis, while the CiF is consistently limited to values below 1 at lower O_2_ concentrations. However, a consistent positive correlation between O_2_ concentration and its effect on the corrosion rate has not been observed. This phenomenon could be attributed to a passivation effect at a certain proportion of O_2_ in the bulk phase of supercritical CO_2_, as noted in several studies: Ayello et al. found 100 ppm of O_2_ had no impact on the corrosion rate in an under-saturated supercritical CO_2_ [4]; Sun et al. conducted a series of experiments and some comparisons in their results revealed a weak correlations between O_2_ presence and the corrosion rate [27]. Two studies attributed such a contradiction to a passivation effect of carbon steels, suggesting that the presence of O_2_ provides better protection for steels at certain concentrations [28,29].

The results of the meta-analysis indicated that the CiF exhibited a tendency related to exposure time. Exposure time exhibited a decreasing trend during the initial 48 h, suggesting a suppressive impact at the early stages of degradation. In contrast, a remarkable increase in the CiF was detected when the exposure time exceeded 72 h.

The above observations align with Hua et al.’s finding in 2016 [16] that the formation of iron hydroxide/oxide corrosion products resulted in passivation/protection of the steel surface at the incipient stage. As the protective layer of hydroxide/oxide developed, the accumulation of internal stress within the FeCO_3_ film led to film detachment and damage, thereby exposing fresh metal to the corrosive substance. Another plausible explanation involves the transition of Fe_2_O_3_ to FeOOH with the exposure time, since goethite possesses a more porous structure and is more susceptible to reactive dissolution. Nonetheless, these results suggest the importance of long-term observation of O_2_ influence on the corrosion rate of supercritical CO_2_.

Apart from the general corrosion rate, the pitting or localized corrosion rate of steels also increased under the influence of O_2_. According to the data reported by previous studies, the extent of pitting corrosion rate on the surface of steels was generally an order of magnitude greater than the general corrosion rate [27,30,31]. Based on a preliminary observation, such an increment in penetration rates was deemed not particularly significant when compared to the general and localized corrosion rates in O_2_-free supercritical CO_2_.

In conclusion, the introduction of O_2_ at a low concentration into the supercritical CO_2_ does not cause a noticeable change in the corrosion rates of carbon steels. Given that the impact is moderate when the concentration of O_2_ exceeds 1%, and this impact of O_2_ becomes increasingly severe over time, anti-corrosion approaches, such as oxidizing inhibitors or other chemical protection methods are recommended.

### 3.2. The Comprehensive Impact of SO_2_ on the Corrosion Rate in Supercritical CO_2_

The corrosion rate of steels in supercritical CO_2_ is also influenced by SO_2_. In a water-saturated supercritical CO_2_, SO_2_ remarkably decreases the pH value of the condensed water and inhibits the dissociation of H_2_CO_3_ [32]. Consequently, the corrosion process is then dominated by SO_2_, which reacts with water and iron to produce FeSO_3_, as illustrated in Equations (10) and (11). In an oxidizing environment, FeSO_3_ can be further oxidized to FeSO_4_.

(10) $$SO_{2}+H_{2}O\to {SO}_{3}^{2-}+2H^{+},$$

(11) $${Fe}^{2+}+{SO}_{3}^{2-}\to FeSO_{3}$$

Macroscopic examinations of corrosion scales on carbon steels revealed the presence of black corrosion scales on their surfaces of the steels [4,27]. Based on the studies conducted by Sun et al. [32] and Choi et al. [33], a double-layer structure was observed in the treated samples. Sun identified that an inner layer of FeSO_4_ containing small amounts of Fe_S_O_3_ and an outer layer of FeOOH were developed on the steel. This finding suggested a strong penetration effect of impurities on the integrity of metals [32,34].

The investigation on the impact of SO_2_ in response to concentration revealed a distinct positive correlation between the concentration of SO_2_ and the general corrosion rate of steels in supercritical CO_2_. An increase in SO_2_ concentration directly led to increased degradation of materials. The above phenomenon could be explained by the structure of the corrosion product FeSO_3_ (or FeSO_4_ in an oxidizing environment). According to the studies by Sun et al. [27,32], the porous and weakly adherent layers of FeSO_3_ or FeSO_4_ failed to block corrosive substance to the metal surface and even created micro-environments that concentrated corrosive species.

Furthermore, the study on the influence of SO_2_ in response to exposure time suggested that SO_2_ acted as a critical substance in accelerating corrosion, and such accumulative damage faded after the initial 24 h, and remained stable thereafter. SO_2_ exerted a continuous detrimental effect on material degradation, suggesting its corrosion-promoting influence persisted throughout the subsequent exposure duration without significant reduction. Collectively, these findings highlight the persistent influence of SO_2_ in supercritical CO_2_ and caution against underestimating its role in corrosion for further industrial applications.

Compared to the general corrosion rate in SO_2_-contaminated supercritical CO_2_, the pitting rate did not reach a significance level [35]. An investigation by Xu et al. [30] reported that one specimen exhibited twice as much of the general corrosion rate in localized attack on the surface of X70 (API 5L Grade X70) after 72 h of exposure to supercritical CO_2_ containing SO_2_ and O_2_. However, this observation is not a positive sign, since the entire surface corroded rapidly.

Regarding the impressive impact factor on corrosion and its consistency through exposure time in supercritical CO_2_, material upgrade for a corrosion environment containing SO_2_ is a critical option. 316L stainless steel and duplex steel with superior corrosion resistance performance are recommended for selection [31]. In addition, proper isolation of SO_2_ with a liner is a cost-effective approach [11].

### 3.3. The Comprehensive Impact of H_2_S on the Corrosion Rate in Supercritical CO_2_

H_2_S is a natural component of natural gas; therefore, the captured CO_2_ from gas plants generally contains a notable amount of H_2_S [36,37]. The introduction of H_2_S to supercritical CO_2_ increases the corrosion rate of steels. A positive correlation is exhibited between the corrosion rate in response to the presence of H_2_S. H_2_S dissociates in free water in the following equations [37]:

(12) $$H_{2}S\to {HS}^{-}+H^{+},$$

(13) $${HS}^{-}\to S^{2-}+H^{+}$$

Despite universal agreement regarding the competitive or synergistic effect of H_2_S on metal degradation in the presence CO_2_, researchers hold different views on the composition and structure of corrosion products. Some early studies [38,39] suggested a protective effect against corrosion at a relatively high partial pressure ratio of H_2_S/CO_2_. Specifically, H_2_S was believed to control the corrosion process through the formation of a dense layer of FeS, which subsequently slowed down the corrosion rate. This tendency was consistent with the response of the corrosion rate analysis, which revealed a reduction in the corrosion rate as H_2_S concentration exceeded 0.1%. Furthermore, it was noted that the concentration of H_2_S regulated corrosion products: FeCO_3_ was discovered at H_2_S concentration below 0.2%, while a firm and dense layer of FeS was found when H_2_S concentration exceeded 0.5% [40,41], The dominant layer of FeS was believed to exert a protective influence on carbon steels.

However, this explanation was not supported by the surface profilometry in the studies conducted over the past decade. A double-layer product was identified on carbon steel in H_2_S-contaminated supercritical CO_2_ [41,42,43]. The outer film of the mushroom-type product consisted of FeS or mackinawite, while the inner film was FeCO_3_. This structure demonstrated that FeS offered poor protection for the integrity of steels. Iron sulfide or mackinawite has also been reported to exhibit electronic conductivity with the ability to generate local galvanic cells at the steel surface, thereby creating electrode potential gradients and accelerating the corrosion process [44]. Wei et al. employed EDS and XRD analyses to reveal the composition of the product. The results indicated that a new layer of FeCO_3_ film was formed on the surface, and the inner film consisted of loose FeCO_3_ and FeS. Wei believed that iron sulfide was the initial product as the chemical adsorption force of HS^−^ was greater than that of HCO_3_^−^. The continuous transportation of Fe^2+^ to the surface induced the formation of FeCO_3_, which elucidated the structure of the product and implied that the presence of H_2_S did not provide adequate protection for the integrity of steels [37].

In supercritical CO_2_, the promoting effect of H_2_S on the corrosion rate of metals intensified with prolonged exposure time. The pattern shaped the process of corrosion: in the initial stage, H_2_S was not active, reacting moderately with metals to form metal sulfides. Over time, the H_2_S-induced corrosion effect was enhanced with the aggregation of FeS or FeS_2_ on the surface of metals. Consequently, despite the decreased CiF when the concentration of H_2_S exceeded 0.1%, the complicated influence on corrosion products implied a possibility that corrosion risks may have been underestimated in early studies.

To summarize, the protective function of the high concentration of H_2_S in supercritical CO_2_ does not contribute to the protection for the integrity of steels, considering the long-term exposure. Duplex stainless steels or nickel-based alloys serve as an appropriate selection to guarantee a safe and reliable service at a medium or high concentration of H_2_S [45,46,47]. Apart from material upgrade, corrosion inhibitors also provide corrosion control through creating a proper isolation between the sulfur-resistant carbon steels and the corrosive agent when the concentration of H_2_S is lower than 0.005% [48].

### 3.4. The Comprehensive Impact of NO_x_ on the Corrosion Rate in Supercritical CO_2_

Despite its low concentration in CO_2_ under most CCS conditions, NO_2_ dramatically accelerates metal degradation in both water-saturated and under-saturated supercritical CO_2_ [40,49,50]. NO_2_ can react with H_2_O to form HNO_3_, a strong acid characterized by its high causticity and oxidizing potential.

(14) $${3NO}_{2}+H_{2}O\to 2{HNO}_{3}+\mathrm{NO}$$

Among the corrosion studies involving NO_2_, Dugstad et al. [49] and Sun et al. [17] discovered that the specimens developed a brown dusty film on their surface after exposure to supercritical CO_2_ with various concentrations of NO_2_. Sun et al. identified that the corrosion products were composed of FeCO_3_ and loose Fe_2_O_3_, with trace amounts of Fe(NO_3_)_3_·9H_2_O. Based on the findings, the corrosion mechanism is explained in the equations shown below:

(15) $${Fe}^{3+}+3{NO_{3}}^{-}\to Fe(NO_{3}{)}_{3},$$

(16) $$4Fe(NO_{3}{)}_{3}\to 2Fe_{2}O_{3}+12NO_{2}+3O_{2}$$

Unlike other impurities that promoted the corrosion process by reacting with steels to inhibit the formation of FeCO_3_, NO_2_ functioned more like a catalyst [51,52]. The corrosion mechanism of NO_2_ made it distinguishable among other impurities, as NO_2_ was not consumed during the degradation process. This conclusion was supported by the remarkable correlation in the meta-analysis, with the impact possessed by NO_2_ being particularly noticeable among impurities at low concentrations and with prolonged exposure times.

It should be noted that part of the data incorporated in the meta-analysis originated from studies utilizing NO as the impurity gas, rather than NO_2_. To ensure comprehensive coverage of the available evidence and to reflect the actual testing conditions reported in the primary literature, the present analysis synthesized the effects of both NO and NO_2_ without distinguishing between them in the effect sizing. This approach is consistent with prevailing practices in the corrosion engineering community, where NO and NO_2_ are often treated interchangeably when both species are recognized as relevant nitrogen-containing impurities in supercritical CO_2_ transmission systems [49]. The treatment aligned with industrial standards that generally group these species in materials performance assessments.

The magnificent impact of NO_x_ on the corrosion acceleration of the carbon steels in supercritical CO_2_ makes it a critical agent in corrosion control. Therefore, strict control of NO_x_ and water in supercritical CO_2_ is the primary option [52].

Resulting from the insufficient number of comparisons included in the datasets, further conclusions could not be statistically supported. Nevertheless, informative findings emerged from the apparent within-group variance: the corrosion rates reported in publications exhibited strong heterogeneity, indicating that diversities in experimental conditions influenced the corrosion process.

### 3.5. Synergistic and Interactive Influence of Impurities on the Corrosion Rate in Supercritical CO_2_

It has been reported that in a complex supercritical CO_2_ system, a complicated and synergistic effect of various impurities intensifies corrosion and degradation of materials [53,54,55]. The influence of impurities imposed on corrosion or degradation of metals is largely dependent on the interactive influence in electrochemistry and the compact level of the corrosive products [2], as the microstructural state and surface film stability influence corrosion resistance [56,57].

Among these impurities, O_2_ and NO_2_ act as strong oxidizing agents, whereas H_2_S functions as a reductive agent; SO_2_ can be either oxidized to SO_3_ or reduced to elemental sulfur and H_2_S. Accordingly, the study on an interactive effect followed an approach governed by the redox properties of O_2_, SO_2_ and H_2_S. NO_2_ was excluded from further study since its significant impact on degradation would inevitably introduce a masking effect (the presence of a strong effect would overshadow or obscure the detection of a weaker but potentially meaningful effect) to the analysis, leading to deviations in corrosion impact factor sizing [52,58].

#### 3.5.1. Synergistic and Interactive Impact of O_2_ and SO_2_ on the Corrosion Rate in Supercritical CO_2_

The presence of O_2_ is believed to alter the corrosion mechanism of metals in water-saturated supercritical CO_2_ with SO_2_, the product, as well as the extent of metal corrosion [31,59]. The introduction of O_2_ to SO_2_ brings interaction of SO_2_, O_2_, and H_2_O, resulting in the formation of a more corrosive sulfuric acid through the oxidation reaction illustrated below:

(17) $$2{SO_{3}}^{2-}+O_{2}\to 2{SO_{4}}^{2-}$$

The mechanism was confirmed in a study where 1000 ppmv of O_2_ was introduced to supercritical CO_2_ containing SO_2_-H_2_O, leading to a change in upper-layer product from FeSO_3_ or FeSO_3_·xH_2_O to a mixture of FeSO_4_·4H_2_O and FeOOH, with trace amounts of FeSO_3_·xH_2_O and FeCO_3_ [27,60].

However, due to the statistically similar CiFs across subgroups and a remarkable *p* value (0.8666) for the impact of SO_2_, as shown in Figure 6a, no significant differences were observed between the subgroups with and without O_2_. The introduction of O_2_ did not change the corrosion rate to a noticeable extent in the SO_2_-H_2_O system, suggesting that O_2_ did not exert a pronounced influence through the oxidation of SO_3_^2−^.

In contrast, the study of the corrosion impact factor of SO_2_ revealed an adverse outcome: the subgroup exposed to O_2_ exhibited a distinctly lower value (11.3968) than the subgroup without O_2_ (12.2711). Notably, the same trend was observed in the study of O_2_ in response to the presence of SO_2_, indicating that O_2_’s corrosive contribution reduced in the presence of SO_2_. Considering the significant difference in CiFs of SO_2_ and O_2_, the dominant role of SO_2_ in the corrosion process effectively masked the contribution of O_2_, leading to its apparent reduction in effect size when both impurities coexist. In a SO_2_-O_2_-CO_2_ system, SO_2_ emerged as the dominant impurity that facilitated the degradation process, while the acceleration of corrosion caused by the presence of O_2_ was statistically underestimated.

Another plausible and explainable interpretation was that the influence of O_2_ may have been diluted by the consumption of O_2_ during the oxidation of SO_3_^2−^, which implied that the presence of O_2_ did not provide a ‘self-governed’ or ‘independent’ contribution to the degradation of steel. However, this hypothesis requires further investigation into the underlying mechanisms involved.

In summary, SO_2_ served as the detrimental substance that initiated steel degradation, compromising the integrity of the FeCO_3_ film, generating porous sulfite and sulfate, and jeopardizing the integrity of the steel. The presence of O_2_ contributed to the propagation of corrosion in a SO_2_-O_2_-CO_2_ system, whereas the overall influence was limited.

#### 3.5.2. Synergistic and Interactive Impact of O_2_ and H_2_S on the Corrosion Rate in Supercritical CO_2_

In pre-combustion processes, H_2_S is typically accompanied by hydrocarbons [21]. Conversely, O_2_ is normally produced in post-combustion processes. The inevitable coexistence of both H_2_S and O_2_ in the bulk phase of supercritical CO_2_ is relevant to coal gasification, waste gas treatment, and biogas utilization, particularly when the CCS industry is extensively developed towards significant reductions in CO_2_ emission.

Compared to O_2_, H_2_S exerts a more substantial effect on corrosion [61]. It is believed that O_2_ contributes to the degradation of steels through an oxidation process that destroys the protective film. At ambient temperature, the simultaneous presence of O_2_ and H_2_S generates elemental sulfur through a redox reaction, as illustrated below [62]:

(18) $$2H_{2}S+O_{2}\to 2S+2H_{2}O$$

The FeS-dominated surface can subsequently be oxidized by O_2_ into a porous and loose layer of Fe(OH)_3_ and sulfur, as described in the following equation:

(19) $$3O_{2}+4FeS+6H_{2}O\to {4Fe(OH)}_{3}+4S$$

Although H_2_S and O_2_ are consumed during the process, the formation of H_2_O generates additional ions. There are different opinions regarding the production of sulfur: Zeng believed that the produced sulfur accelerated the dissolution of Fe on the surface and caused further metal degradation through the formation of FeS [63]. Sun et al. [27] identified a significant amount of sulfur in an inner-layer product, suggesting that the formation of FeS through a solid state reaction was not as easy as anticipated.

The meta-analysis reveals distinct patterns in the individual and combined effects of H_2_S and O_2_. As shown in Figure 7a,b, the CiF values for H_2_S and O_2_ solely approximate their respective overall CiF contributions of 0.8729 and 1.9185. This observation suggested that, in the absence of the other impurity, each impurity exerted a relatively independent corrosion influence. The CiF of H_2_S in the presence of O_2_ reached 3.5475, which markedly exceeded the sum of their individual effect sizes (2.7914). This deviation from simple addition indicated the presence of a synergistic interaction between the two corrosive species. In other words, the combined corrosive action of H_2_S and O_2_ was not merely the cumulative result of two independent processes, but rather an amplified effect in the supercritical CO_2_ environment.

This synergistic behavior is further supported by the analysis of O_2_’s effect in the presence of H_2_S. Under H_2_S-containing conditions, the effect size of O_2_ (1.8470) was significantly greater than that observed under O_2_-alone conditions, with a synergistic index of approximately 1.31 (calculated as the combined effect divided by the expected effect). This value indicated a statistically robust interactive corrosion behavior in the system. However, when H_2_S was present, the individual effect of O_2_ on corrosion was lower than the combined influence of both O_2_ and H_2_S. These results indicated that H_2_S serves as the primary factor governing corrosion. Consequently, the coexistence of O_2_ and H_2_S resulted in more complex products and surface morphology and contributed to a prompt but non-detrimental degradation of carbon steels [61,64]. In an H_2_S-O_2_-CO_2_ system where chemical inhibitors are applied for pipeline protection, oxygen scavengers are recommended to prevent damage to the protective layer and the dissolution of Fe on the surface.

The *p* values between groups of 0.1536 and 0.2680 indicated a notable but not robust difference between the two subgroups. While industry widely recognizes that product and surface complexity accelerates metal degradation via microgalvanic interactions [65], whether sulfur or FeS formation contributes to the overall weight change in steels remains unclear [66,67]. This mechanism requires further investigation that supports the synergistic and interactive influence of H_2_S and O_2_.

## 4. Materials and Methods

### 4.1. Data Collection

Journal articles published between 2010 and 2025 that reported the corrosion rates of steels in response to impurities in supercritical CO_2_ were reviewed using the Web of Science database. The search terms were “Supercritical CO_2_ (Topic) and corrosion (Topic) and impurities (Abstract) and 2010–2025 (Year Published).” Data were collected from studies that met the following criteria: (1) The selected reports must include at least one variable related to the concentration of H_2_S, SO_2_, NO_x_, and O_2_ under either water-saturated or under-saturated supercritical CO_2_ conditions. (2) The average or general corrosion rate (mm∙year-1) was utilized as an indicator of the degree of influence on corrosion; the general corrosion rate measured through the weight loss tests and resistance relaxation methods were both accepted. (3) The sample replicate numbers were derived from the studies that reported the number of replicates.

The literature search identified a total of 109 publications, among which 38 were excluded after abstract screening due to irrelevance and duplication. After full-text review of the remaining 71 publications, 56 were discarded because their data did not meet the criteria outlined in the previous paragraph. Additionally, 12 individual publications (from citations or reviews) from other sources were included in the analysis. Consequently, a total of 27 published papers with 149 individual studies were retained for analysis. The flow diagram of study selection is plotted in Figure 8. The complete database contained four impurities, namely, O_2_, SO_2_, H_2_S, and NO_x_.

Potential sources of heterogeneity were explored based on two factors, i.e., concentration of impurities and exposure time. The entire dataset was divided to illustrate the responses of the general corrosion rate to the existence of impurities in supercritical CO_2_ under various classification factors. The concentrations of impurities were divided into three categories: 0.002% to 0.05% (inclusive of 0.05%), 0.05 to 0.1% (inclusive of 0.1%), and above 0.1%. The exposure time was classified into three categories: below 24 h (inclusive of 24 h), 24 h to 72 h (inclusive of 72 h), and above 72 h.

The synthetic or interactive influence of two impurities was investigated through the subgroup analysis. Combinations were selected based on the respective redox characteristics of impurities.

### 4.2. Meta-Analysis

Data were collected from the publications shown in Supplementary Table S1 [5,16,17,24,26,27,29,30,32,33,35,36,40,41,49,52,59,66,67,68,69,70,71,72,73,74,75]. The meta-analysis method applied in this study followed well-established criteria conducted within the field of ecology [23,76,77]. Generally, a meta-analysis incorporates the replication-based weighting function, which depends on the integrity of reported standard deviations in the datasets. Unfortunately, the corrosion rates reported in most studies were not derived from replicated data; even when the corrosion rates were based on repeatable experiments, standard deviations were typically not provided in publications. Therefore, it is impossible to evaluate the effect sizes directly in the absence of standard deviations.

Data from the publications were reprocessed according to the following principles:

The corrosion rate from publications based on replicating experiments with replicate numbers presented was recorded for meta-analysis.

For publications without replicates presented, a minimum of two replicates was assumed.

Standard deviation (SD) was then calculated through the equation:

(20) $${SD}_{t}=\frac{{x_{t}}^{2}{CV}^{2}}{n_{t}}$$

(21) $${SD}_{c}=\frac{{x_{c}}^{2}{CV}^{2}}{n_{c}}$$

where SD_t_ and SD_c_ were the standard deviations of the mean values of the general corrosion rate of the experiment and control groups, respectively; CV represented the coefficient of variation, which was taken as 0.3 in this study, based on an assumption of moderate relative variability in the datasets [23,78,79]. The values n_t_ and n_c_ indicated the number of replicates for the influence and control groups, while x_t_ and x_c_ corresponded to the general corrosion rate of the influence and control groups, respectively.

To assess the robustness of the application, a sensitivity analyses using alternative CV assumptions (0.2 and 0.4) was conducted. The results are presented in Supplementary Material as Figures S1 and S2 and Tables S4 and S5.

The response ratio (RR) method was used to assess the influence of impurities on corrosion in water-saturated or under-saturated supercritical CO_2_. RR was calculated as the natural logarithm of the ratio of the influence group (x_t_) to the control group (x_c_) using the following equation:

(22) RR=lnxtxc[mmyear−1]=lnxt−ln(xc)

The results were transformed to represent the physical concept of the corrosion impact factor (CiF) [27] using the formula below:

(23) $$Corrosion\;Impact\;Factor\;on\;the\;general\;corrosion\;rate=e^{RR}-1$$

Notably, the findings of this meta-analysis regarding the corrosion rate align with the physical concept of the corrosion impact factor (CiF) in terms of core connotation and variation trend. As a dimensionless engineering index to quantify the extent of degradation, CiF is defined as the difference in the corrosion rate between the target environment and the standard reference environment divided by the corrosion rate at the standard reference. This definition captures the relative changes in outcomes resulting from variations in the corrosive environment. Thus, Equations (22) and (23) present precise and appropriate observations in this meta-analysis that elucidate the influence of impurities on the corrosion rate as reflected in the CiF result.

The mean effect sizes and 95% confidence intervals (CIs) were generated using software MetaWin Version 2.1 (Sinauer Associates, Inc., Sunderland, MA, USA). To analyze the influence of variables on the impurity-induced effect on the corrosion rate, a bootstrapping procedure with 4999 iterations was performed.

The data collected were derived from studies that varied in experimental conditions, including temperature, pressure, exposure time, and types of carbon steels. The inter-group variance was not negligible. A random-effects model was employed to implement the analysis in evaluating the effect size [80]. Under the random-effects model, the overall effect estimate is a weighted average of the individual study results, where the weights incorporate both the within-study variance and the between-study variance.

Subgroup analysis was performed to investigate the possible corrosion impact factors. In examining the correlations between the effect sizes of continuous variables, a variable is considered significant when *p* < 0.05, which serves as a standard criterion. However, resulting from the limited number of corrosion studies, a lower *p* value confirms the true effect size difference, while a *p* value exceeding 0.05 requires further validation through Q statistics [80].

The fail-safe analysis was performed using Rosenthal’s method to determine the risk of sampling bias in the literature search, and the criteria for reliable results are shown in the following equation:

N > 5K + 10(24)

where N represents the fail-safe number and K is the number of comparisons in the meta-analysis. Despite the small sample size of corrosion-related studies, which suggested that the results were unlikely to be inflated by a single study, the fail-safe analysis still suffered from bias when publication bias is substantial. The results are presented in Supplementary Tables S2 and S3 for reference.

## 5. Conclusions

Impurities such as O_2_, SO_2_, H_2_S, and NO_x_ accelerate the corrosion rate and pose a serious threat to the integrity of carbon steels in supercritical CO_2_ systems where water has not been completely removed. The study findings are summarized as follows:

The introduction of O_2_ to supercritical CO_2_ mildly promotes steel degradation when the concentration of O_2_ is above 0.1%. No significant response is observed in the general corrosion rate when the concentration of O_2_ is below 0.1%.

H_2_S is an aggressive contaminant that exerts a profound influence on the corrosion rate of carbon steels. A synergistic effect is observed when H_2_S and O_2_ coexist in supercritical CO_2_, leading to accelerated degradation.

SO_2_ has a detrimental and sustained influence on material degradation, with the corrosion-promoting effect persisting throughout the duration of exposure and exhibiting no significant attenuation. The synergistic influence on the corrosion rate in supercritical CO_2_ of SO_2_ and O_2_ is statistically limited.

NO_2_ in supercritical CO_2_ plays a vital role in corrosion propagation. Distinct from H_2_S, SO_2_, and O_2_ which are consumed during the degradation process, NO_2_ is replenished in the system at the same rate as it is consumed.

The limitations of this meta-analysis should be acknowledged. In addition to the impurities discussed in this article, parameters such as water concentration, temperature, pressure, steel grade, surface condition, and the presence other impurities such as hydrogen and methane also influence the degradation and precipitation kinetics of metals. Furthermore, it remains unclear whether the products are stable or continue to propagate under the same conditions, and the corrosion behavior requires further investigation. Finally, subgroup analyses did not reveal credible evidence for the sources of heterogeneity. Future studies aimed at developing experiments that accurately evaluate the diversity of factors involved in determining the threat of corrosion and developing a comprehensive pipeline integrity management strategy are warranted.

## Figures and Tables

**Figure 1:**
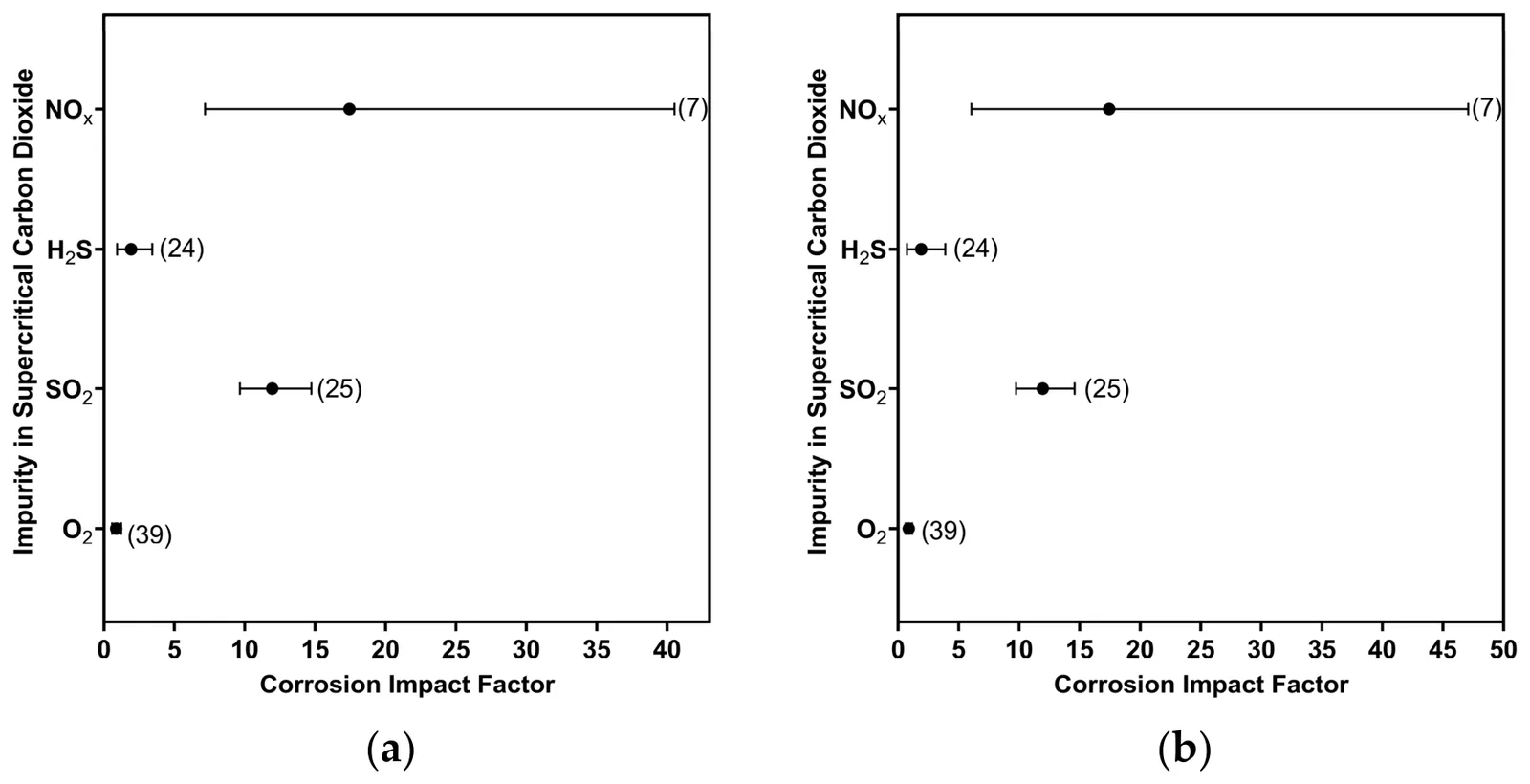
The influence on the general corrosion rate of carbon steels in supercritical CO_2_ to O_2_, SO_2_, H_2_S, and NO_x_ across subgroups of (**a**) concentration and (**b**) exposure time.

**Figure 2:**
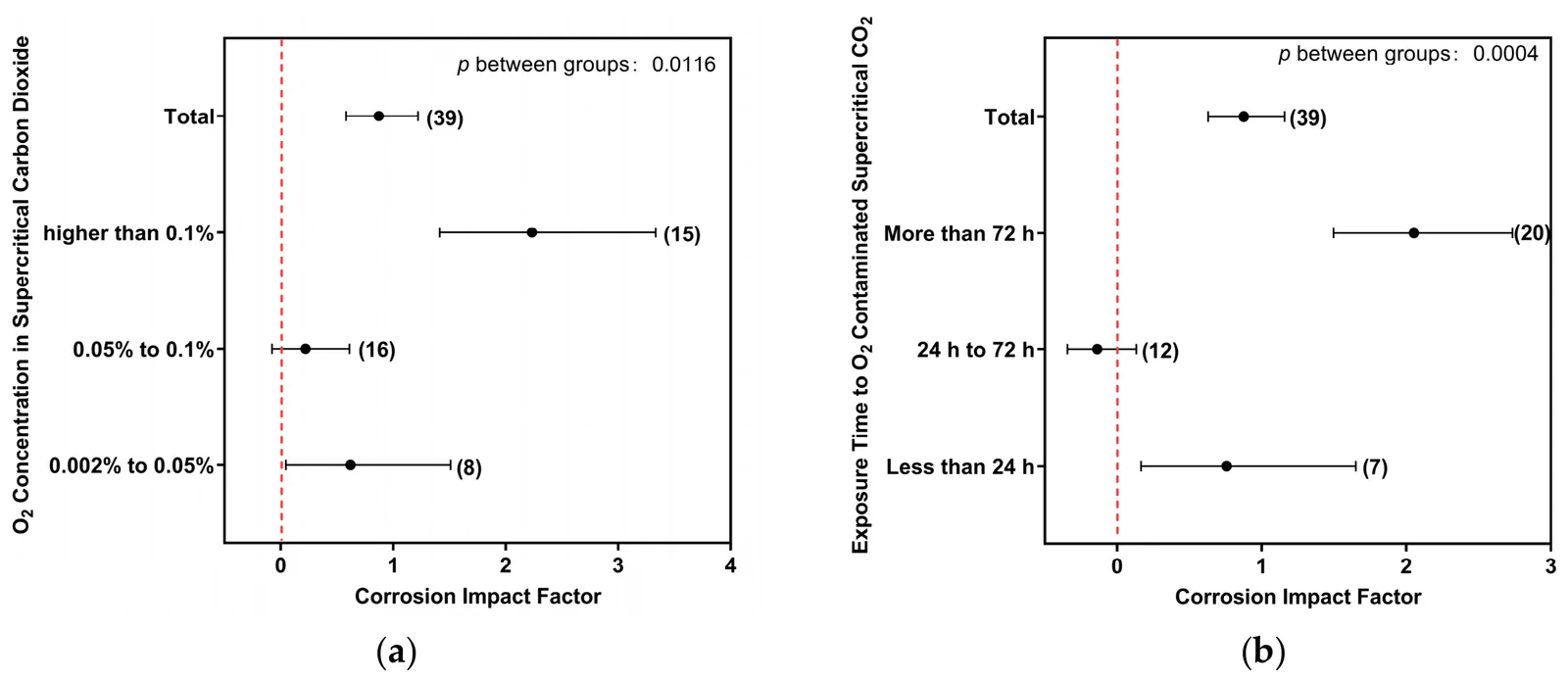
The influence on the general corrosion rate of carbon steels in supercritical CO_2_ to O_2_ at different (**a**) concentrations and (**b**) exposure times.

**Figure 3:**
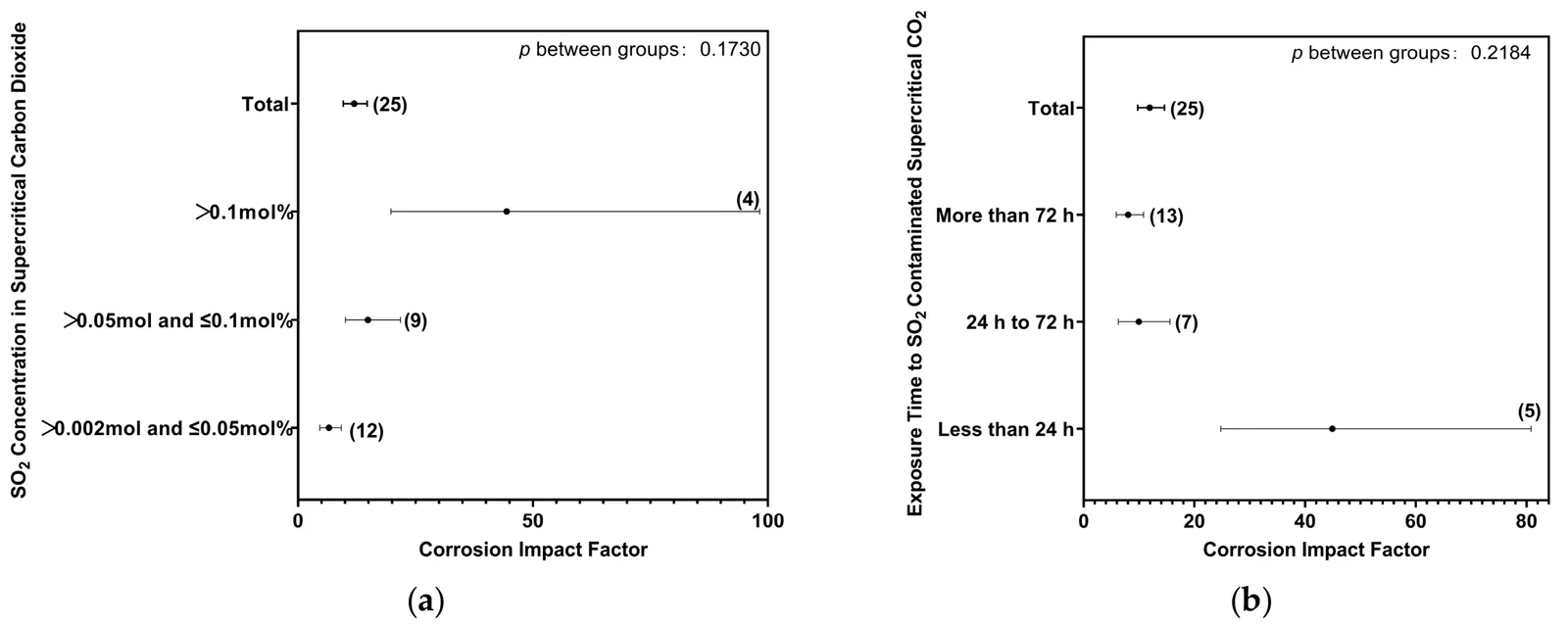
The influence on the general corrosion rate of carbon steels in supercritical CO_2_ to SO_2_ at different (**a**) concentrations and (**b**) exposure times.

**Figure 4:**
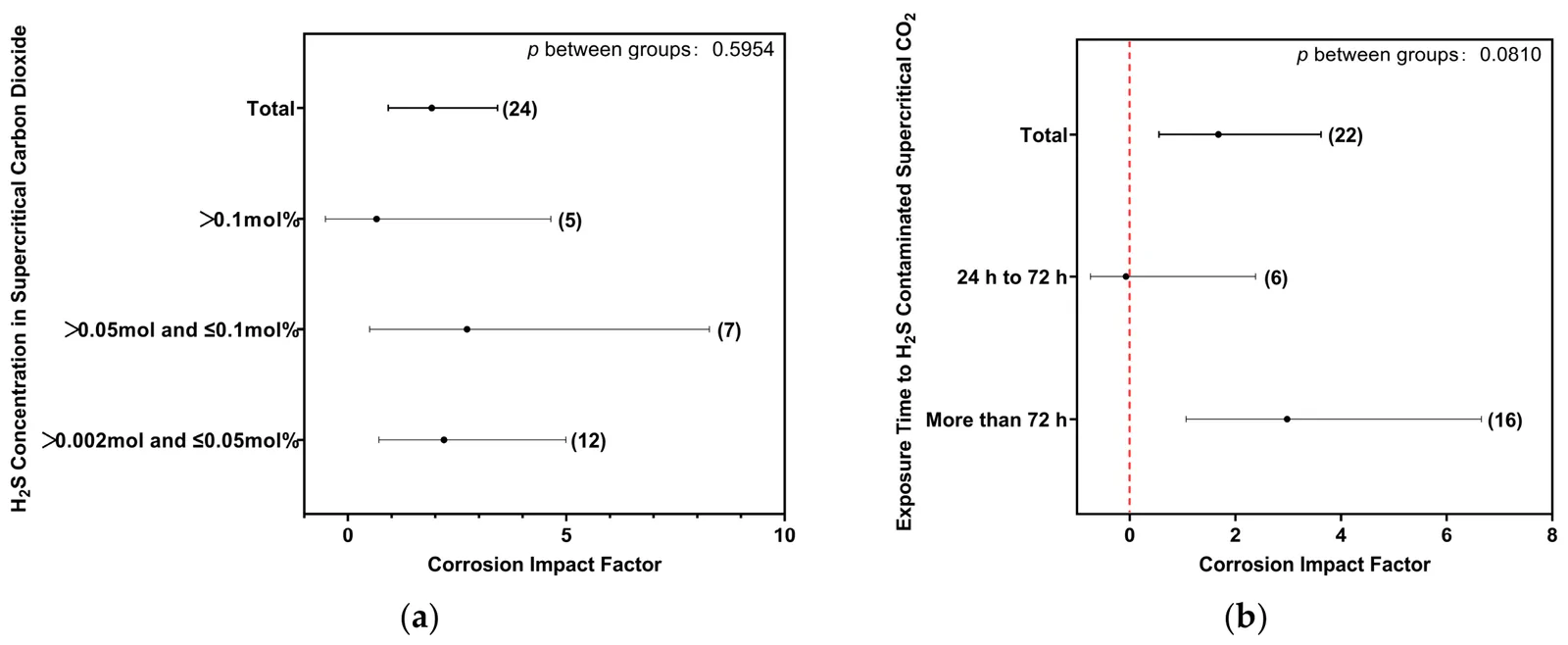
The influence on the general corrosion rate of carbon steels in supercritical CO_2_ to H_2_S at different (**a**) concentrations and (**b**) exposure times.

**Figure 5:**
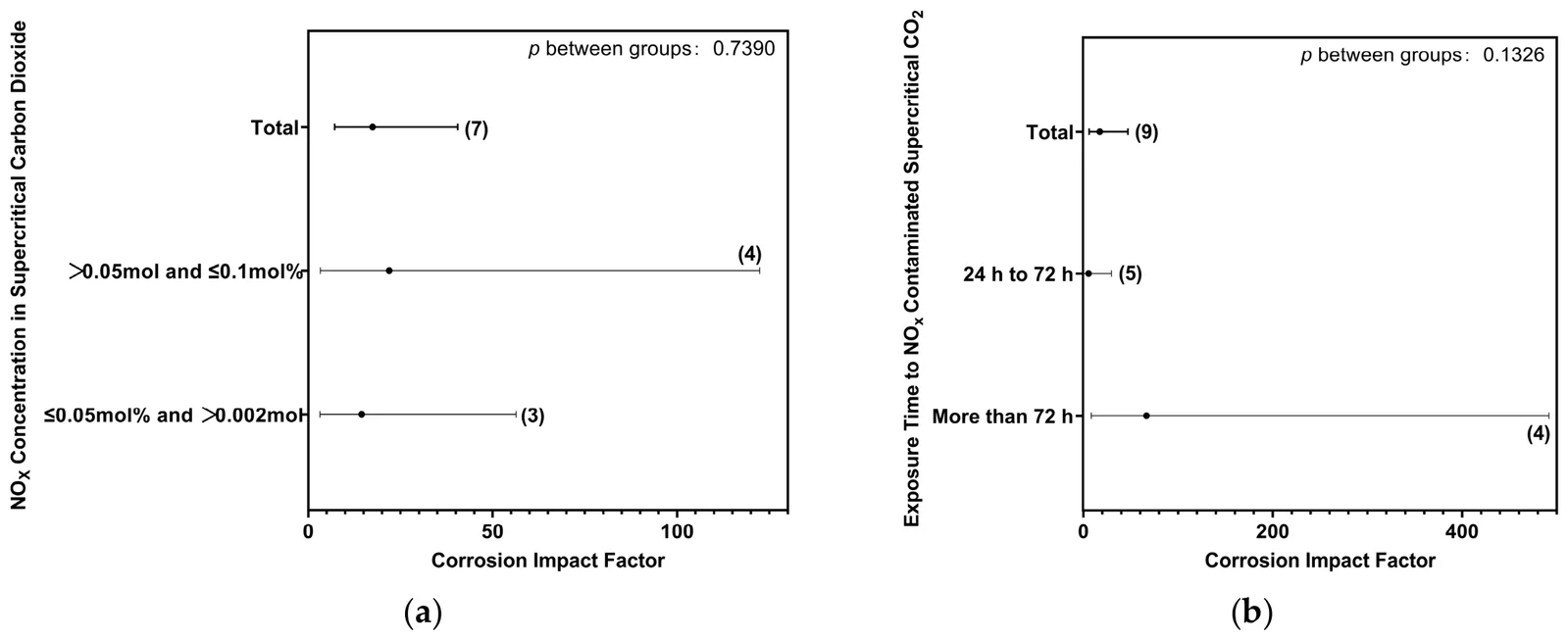
The influence on the general corrosion rate of carbon steels in supercritical CO_2_ to NO_x_ at different (**a**) concentrations and (**b**) exposure times.

**Figure 6:**
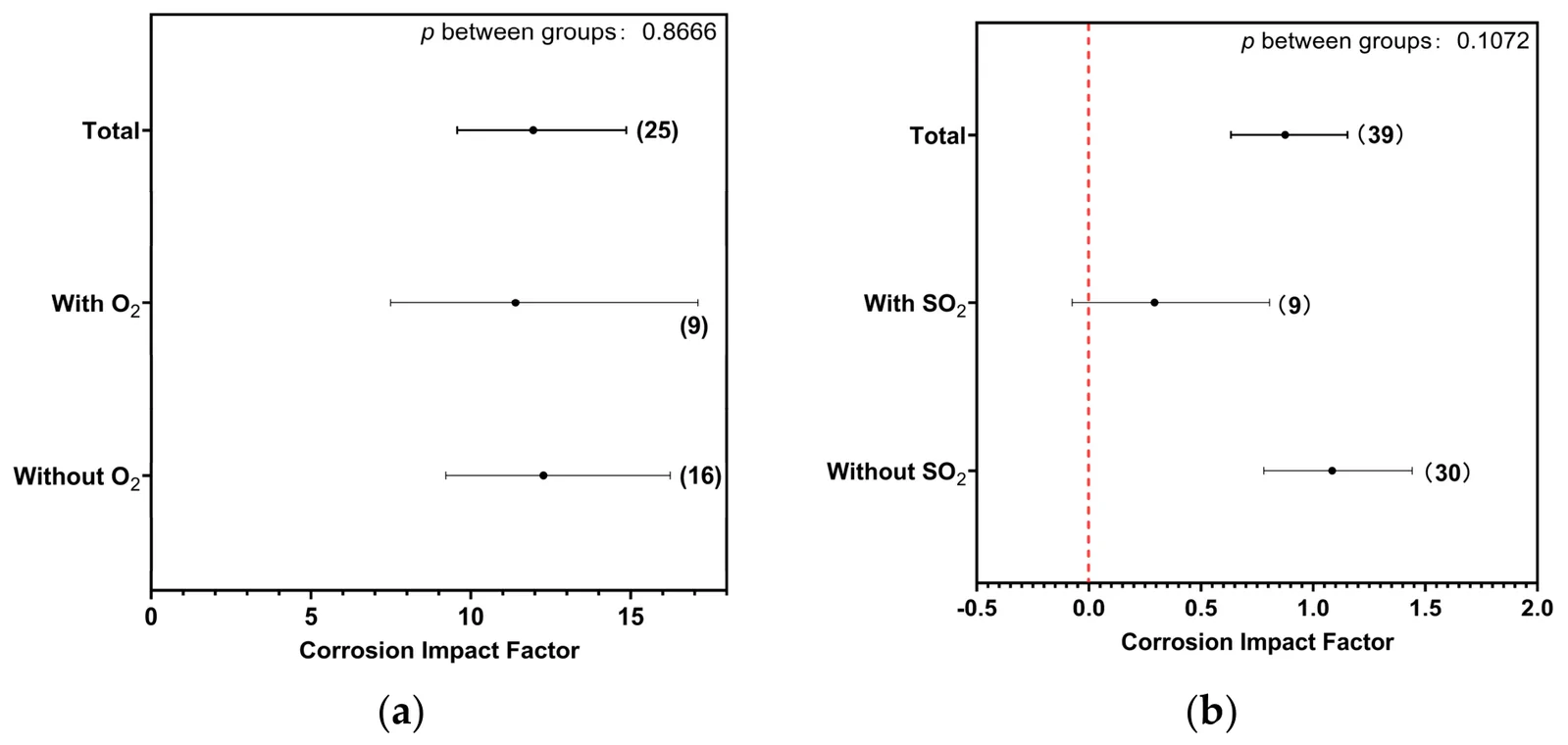
The influence on the general corrosion rate of carbon steels in supercritical CO_2_ in response to (**a**) SO_2_ and (**b**) O_2._

**Figure 7:**
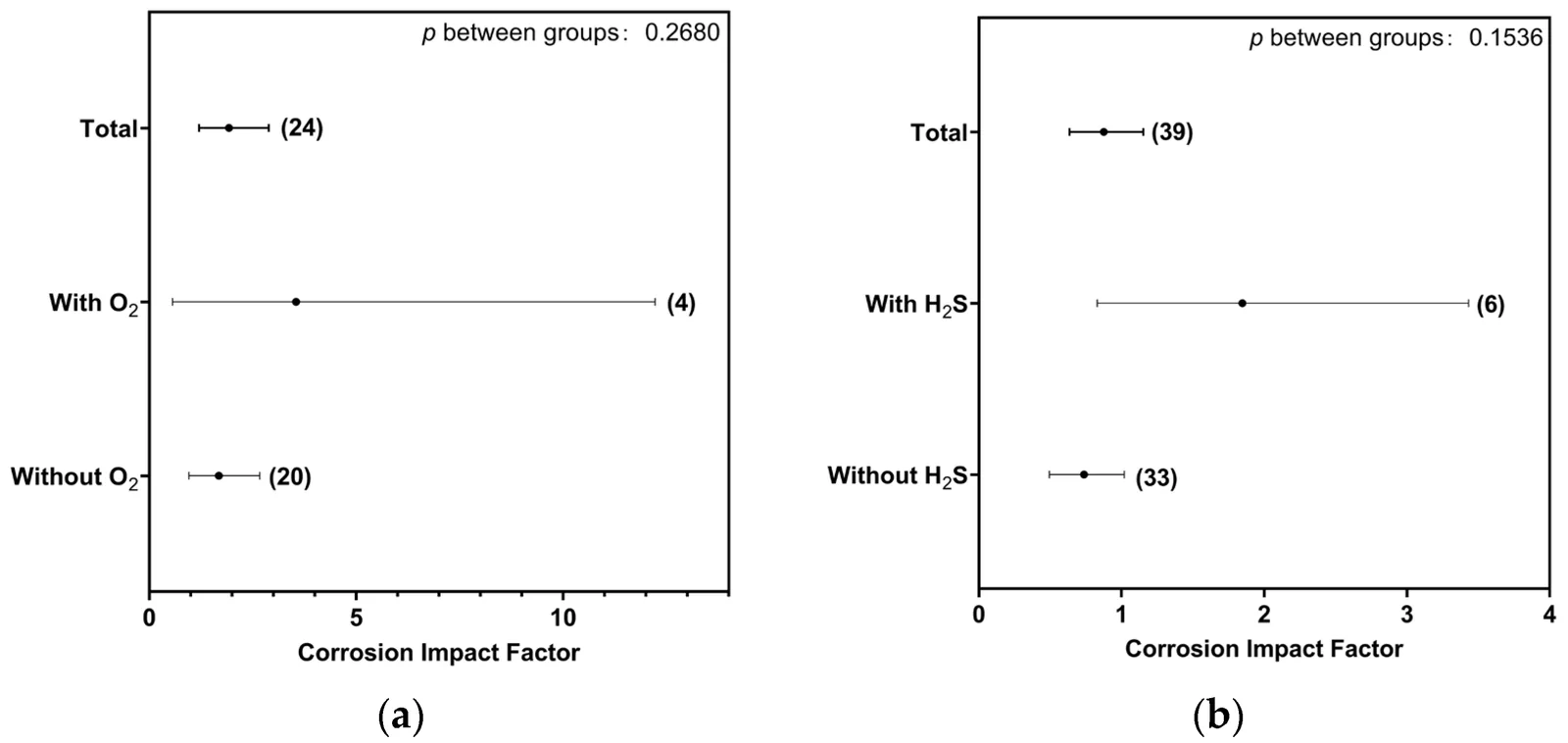
The influence on the general corrosion rate of carbon steels in supercritical CO_2_ in response to (**a**) H_2_S and (**b**) O_2._

**Figure 8:**
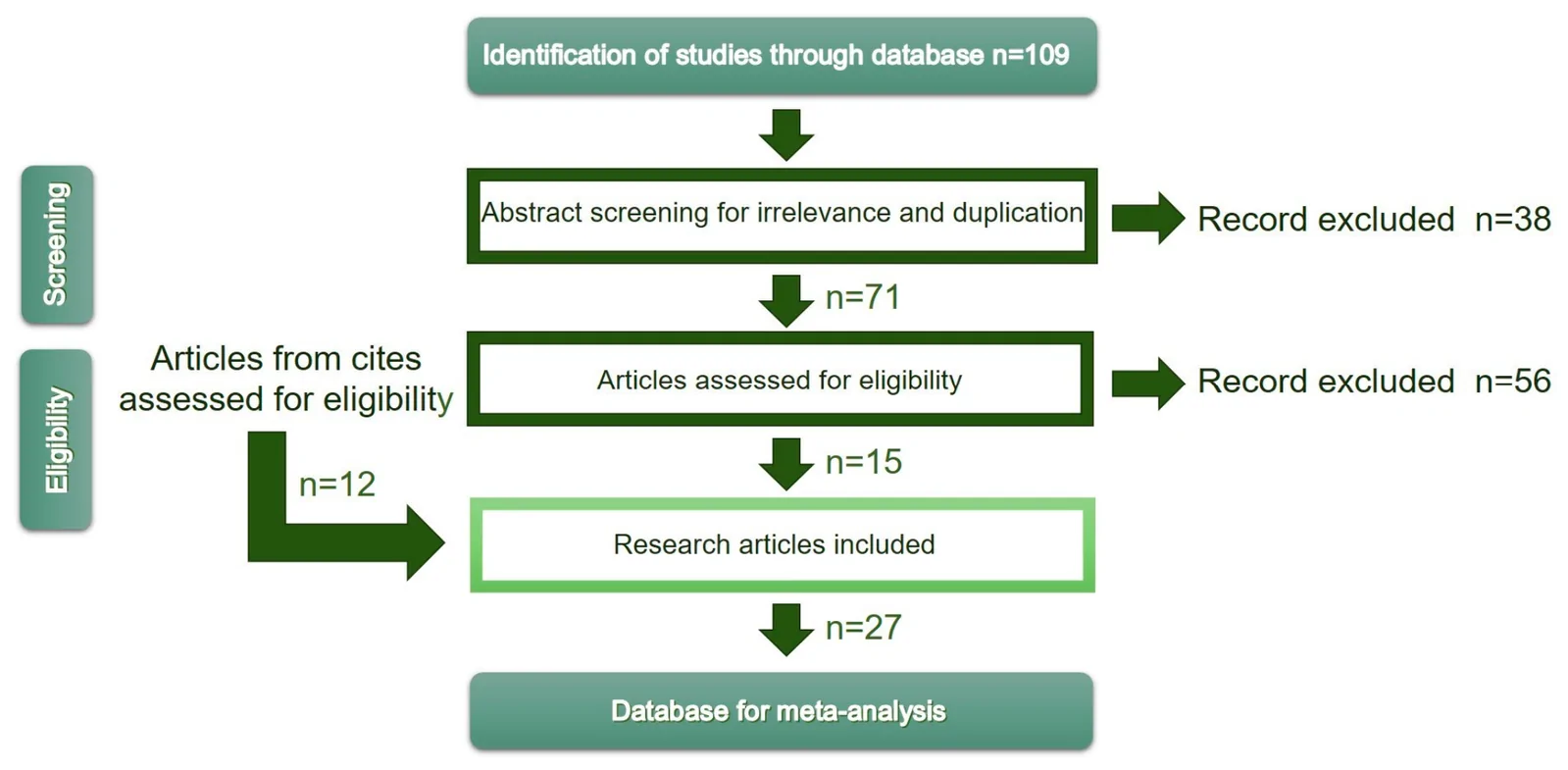
Flowchart of the literature search and inclusion process.

## Data Availability

The data presented in this study are available on request from the corresponding author upon reasonable request, as all input data are extracted from published literature subject to third-party copyright. The shared dataset includes only processed effect sizes and analysis intermediates for verification and replication purposes; original data of individual included studies must be requested from their respective original authors.

## Supplementary Materials

The following supporting information can be downloaded at https://www.mdpi.com/article/10.3390/molecules31203631/s1, Table S1. The list of publications used in this meta-analysis; Table S2. The meta-analysis results of impurities on the corrosion rates in water-saturated and under-saturated supercritical CO_2_ (categorized by concentration). Table S3. The meta-analysis results of impurities on the corrosion rates in water-saturated and under-saturated supercritical CO_2_ (categorized by exposure time). Table S4. The overall impact factor and 95% confidence intervals for different CV values of the influence of impurities on corrosion in supercritical CO_2_ (categorized by concentration); Table S5. The overall impact factor and 95% confidence intervals for different CV values of the influence of impurities on corrosion in supercritical CO_2_ (categorized by exposure time). Figure S1. The influence on the general corrosion rate of carbon steels in supercritical CO_2_ to O_2_, SO_2_, H_2_S, and NO_x_ across subgroups of concentration at different CV. Figure S2. The influence on the general corrosion rate of carbon steels in supercritical CO_2_ to O_2_, SO_2_, H_2_S, and NO_x_ across subgroups of exposure time at different CV.

## Abbreviations

The following abbreviations are used in this manuscript:

CCS	carbon capture and storage
CI	confidence intervals
CiF	corrosion impact factor
CV	coefficient of variation
EDS	energy dispersive spectroscopy
Fe(NO_3_)_3_	iron nitrate
Fe_2_O_3_	iron oxide
FeCO_3_	iron carbonate
FeOOH	iron oxyhydroxide
FeS	iron sulfide
FeSO_3_	iron sulfite
FeSO_4_	iron sulfate
H_2_	hydrogen
H_2_CO_3_	carbonic acid
H_2_S	hydrogen sulfide
HNO_3_	nitric acid
NO_x_	nitrogen oxides
O_2_	oxygen
RR	response ratio
SD	standard deviation
SEM	scanning electron microscopy
SO_2_	sulfur dioxide
SO_3_	sulfur trioxide
XRD	X-ray diffraction
