# Supplementary material for: Weighted Analysis of Corrosion-Inducing Impurities in Supercritical CO2 Transportation System
Source: Molecules. 2026 Oct 12;31(20):3631. doi: 10.3390/molecules31203631 (PMC13707878; doi:10.3390/molecules31203631)
Supplement: Supplementary file 1 [file molecules-31-03631-s001.zip › molecules-4583117-supplementary.pdf]

Table S1:The list of publications used in this meta-analysis

No.	References
1	Choi, Y.S.; Nesic, S.; Young, D. Effect of impurities on the corrosion behavior of CO_2 transmission pipeline steel in supercritical CO2-water environments. *Environ. Sci. Technol.* **2010**, *44*, 9233–9238. https://doi.org/10.5006/c2010-10196.
2	Choi, Y.S.; Nešic, S. Effect of Water Content on the Corrosion Behavior of Carbon Steel in Supercritical CO_2 Phase with Impurities. In Proceedings of the NACE International Corrosion Conference, Houston, TX, USA, 13-17 March 2011.
3	Choi, Y.S.; Hassani, S.; Vu, T.N.; Nesic, S.; Abas, A.Z.B. Effect of H_2S on the corrosion behavior of pipeline steels in supercritical and liquid CO_2 environments. *Corrosion* **2016**, *72*, 999–1009. https://doi.org/10.5006/2026.
4	Xiang, Y.; Wang, Z.; Xu, C.; Zhou, C.; Li, Z.; Ni, W. Impact of SO_2 Concentration on the Corrosion Rate of X70 Steel and Iron in Water-Saturated Supercritical CO_2 Mixed with SO_2. J. *Supercrit. Fluids* **2011**, 58, 286-294. https://doi.org/10.1016/j.supflu.2011.06.007.
5	Paschke, M.; Kather, A. Corrosion of Pipeline and Compressor Materials Due to Impurities in Separated CO_2 from Fossil-Fuelled Power Plants. *Energy Procedia* **2012,** 23, 207-215.
6	Hua, Y.; Barker, R.; Neville, A. The effect of O_2 content on the corrosion behaviour of X65 and 5Cr in water-containing supercritical CO_2 environments. *Appl. Surf. Sci.* **2015**, *356*, 499–511. https://doi.org/10.1016/j.apsusc.2015.08.116.
7	Hua, Y.; Barker, R.; Neville, A. Understanding the influence of SO_2 and O_2 on the corrosion of carbon steel in water-saturated supercritical CO_2. *Corrosion* **2015**, *71*, 667–683. https://doi.org/10.5006/c2015-05832.
8	Hua, Y.; Barker, R.; Neville, A. The influence of SO_2 on the tolerable water content to avoid pipeline corrosion during the transportation of supercritical CO_2. *International Journal of Greenhouse Gas Control* **2015**, 37, 412-423. https://doi.org/10.1016/j.ijggc.2015.03.031.
9	Sun, C.; Wang, Y.; Sun, J.; Lin, X.; Li, X.; Liu, H.; Cheng, X. Effect of impurity on the corrosion behavior of X65 steel in water-saturated supercritical CO_2 system. *J. Supercrit. Fluids* **2016**, *116*, 70–82. https://doi.org/10.1016/j.supflu.2016.05.006.
10	Sun, C.; Sun, J.B.; Wang, Y.; Lin, X.Q.; Li, X.D.; Cheng, X.K.; Liu, H.F. Synergistic effect of O_2, H_2S and SO_2 impurities on the corrosion behavior of X65 steel in water-saturated supercritical CO_2 system. *Corros. Sci.* **2016**, *107*, 193–203. https://doi.org/10.1016/j.corsci.2016.02.032.
11	Sun, J.; Sun, C.; Zhang, G.; Li, X.; Zhao, W.; Jiang, T.; Liu, H.; Cheng, X.; Wang, Y. Effect of O_2 and H_2S impurities on the corrosion behavior of X65 steel in water-saturated supercritical CO_2 system. *Corros. Sci.* **2016**, *113*, 104–117. https://doi.org/10.1016/j.corsci.2016.02.017.
12	Wei, L.; Zhang, Y.; Pang, X.; Gao, K. Corrosion behaviors of steels under supercritical CO_2 conditions. *Corros. Rev.* **2015**, *33*, 151–174. https://doi.org/10.1515/corrrev-2014-0067.
13	Sun, C.; Sun, J.; Wang, Y.; Sui, P.; Lin, X.; Liu, H.; Cheng, X.; Zhou, M. Effect of impurity interaction on the corrosion film characteristics and corrosion morphology evolution of X65 steel in water-saturated supercritical CO_2 system. *Int. J. Greenh. Gas Control* **2017**, *65*, 117–127. https://doi.org/10.1016/j.ijggc.2017.09.002.
14	Xu, M.; Zhang, Q.; Wang, Z.; Liu, J.; Li, Z. Effect of High-Concentration O2 on Corrosion Behavior of X70 Steel in Water-Containing Supercritical CO_2 with SO_2. *Corrosion* **2017**, 73, 290-302. https://doi.org/10.5006/2175.
15	Tang, Y.; Guo, X.P.; Zhang, G.A. Corrosion behaviour of X65 carbon steel in supercritical-CO_2 containing H_2O and O_2 in carbon capture and storage (CCS) technology. *Corros. Sci.* **2017**, *118*, 118–128. https://doi.org/10.1016/j.corsci.2017.01.028.
16	Hua, Y.; Jonnalagadda, R.; Zhang, L.; Neville, A.; Barker, R. Assessment of general and localized corrosion behavior of X65 and 13Cr steels in water-saturated supercritical CO_2 environments with SO_2/O_2. *Int. J.*

No.	References
	Greenh. Gas Control **2017**, *64*, 126–136. https://doi.org/10.1016/j.ijggc.2017.07.012.
17	Sun, J.; Sun, C.; Wang, Y. Effects of O_2 and SO_2 on water chemistry characteristics and corrosion behavior of X70 pipeline steel in supercritical CO2 transport system. *Ind. Eng. Chem. Res.* **2018**, *57*, 2365–2375. https://doi.org/10.1021/acs.iecr.7b04870.
18	Xu, M.H.; Zhang, Q.; Wang, Z.; Liu, J.M.; Li, Z. Cross impact of CO_2 phase and impurities on the corrosion behavior for stainless steel and carbon steel in water-containing dense CO_2 environments. *Int. J. Greenh. Gas Control* **2018**, *71*, 194–211. https://doi.org/10.1016/j.ijggc.2018.02.011.
19	Sun, J.L. Insights into the interfacial process in electroless Ni-P coating on supercritical CO_2 transport pipeline as relevant to carbon capture and storage. ACS Appl. *Mater. Interfaces* **2019**, *11*, 16243–16251. https://doi.org/10.1021/acsami.9b03623.
20	Teeter, L.; Repukaitis, R.; Huerta, R.P.; Oleksak, R.B. Effect of O_2 on the Long-Term Operation and Corrosion of Steel X65 in CO_2-H_2O Environments for Direct Supercritical CO_2 Power Cycle Applications. *J. Supercrit. Fluids* **2019**, 143, 274-284. https://doi.org/10.1016/j.supflu.2019.04.007.
21	Wang, W.H.; Shen, K.L.; Tang, S.; Shen, R.Q.; Parker, T.; Wang, Q.S. Synergistic effect of O_2 and SO_2 gas impurities on X70 steel corrosion in water-saturated supercritical CO_2. *Process Saf. Environ. Prot.* **2019**, *130*, 57–66. https://doi.org/10.1016/j.psep.2019.07.017.
22	Dugstad, A.; Halseid, M.; Morland, B.R. Effect of SO_2 and NO_2 on corrosion and solid formation in dense phase CO_2 pipelines. *Energy Procedia* **2013**, *37*, 2877–2887. https://doi.org/10.1016/j.egypro.2013.06.173.
23	Li, K.Y.; Zeng, Y.M.; Luo, J.L. Influence of H_2S on the general corrosion and sulfide stress cracking of pipelines steels for supercritical CO_2 transportation. *Corros. Sci.* **2021**, *190*, 109639. https://doi.org/10.1016/j.corsci.2021.109639.
24	Sun, C.; Ding, T.; Sun, J.; Lin, X.; Zhao, W.; Chen, H. Insights into the effect of H_2S on the corrosion behavior of N80 steel in supercritical CO_2 environment. *J. Mater. Res. Technol.* **2023**, *26*, 5462–5477. https://doi.org/10.1016/j.jmrt.2023.08.277.
25	Collier, J.; Papavinasam, S.; Li, J.; Shi, C.; Gravel, J. P. Effect of impurities on the corrosion performance of steels in supercritical carbon dioxide: Optimization of experimental procedure. NACE - International Corrosion Conference Series 2013.
26	Jock; Brown; Brit; Graver; Egil; Gulbrandsen; Arne; Dugstad; Bjørn; Morland. Update of DNV Recommended Practice RP-J202 with Focus on CO_2 Corrosion with Impurities. *Energy Procedia* **2014**, 63, 2432-2441. https://doi.org/10.1016/j.egypro.2014.11.265.
27	Simonsen, K.R.; Goebel, J.; Hansen, D.S.; Pedersen, S. The influence of temperature, H_2O, and NO_2 on corrosion in CO_2 transportation pipelines. *Process Saf. Environ. Prot.* **2025**, *198*, 107190. https://doi.org/10.1016/j.psep.2025.107190.

Table S2: The meta-analysis results of impurities on the corrosion rates in water-saturated and under-saturated supercritical CO_2 (categorized by concentration)

No.	Impurity	Qtotal	Sqrt Pooled Variance	Mean Effect Size	95%CI	Bootstrap CI	Fail-safe N
1	O_2	91.6002	0.5229	0.8729	0.5797 to 1.2207	0.4522 to 1.4123	758.1

2	SO_2	210.0774	0.4686	11.9454	9.6524 to 14.7319	6.6335 to 21.1016	6768.0
3	H_2S	48.1484	0.9855	1.9185	0.9243 to 3.4264	0.6683 to 4.1052	226.3
4	NO_x	24.0564	1.0561	17.4306	7.1822 to 40.5153	4.8284 to 55.5390	218.8

Table S3: The meta-analysis results of impurities on the corrosion rates in water-saturated and under-saturated supercritical CO_2 (categorized by exposure time)

No.	Impurity	Qtotal	Sqrt Pooled Variance	Mean Effect Size	95%CI	Bootstrap CI	Fail-safe N
1	O_2	135.4050	0.4298	0.8754	0.6300 to 1.1577	0.4597 to 1.4196	1139.2
2	SO_2	231.4696	0.4460	11.9411	9.7477 to 14.5821	6.5166 to 21.1489	7458.7
3	H_2S	30.9890	1.2290	1.9156	0.7346 to 3.9007	0.6623 to 4.0712	136.9
4	NO_x	17.2577	1.2471	17.4348	6.0669 to 47.0888	5.0299 to 56.6837	154.4

Table S4: The overall impact factor and 95% confidence intervals for different CV values of the influence of impurities on corrosion in supercritical CO_2 (categorized by concentration)

No.	Impurity	CV	Mean Effect Size	95%CI
1	O_2	0.2	0.8678	0.5763 to 1.2132

No.	Impurity	CV	Mean Effect Size	95%CI
		0.3	0.8729	0.5797 to 1.2207
		0.4	0.8817	0.5853 to 1.2336
2	SO_2	0.2	11.9738	9.6886 to 14.7477
		0.3	11.9454	9.6524 to 14.7319
		0.4	11.8415	9.5422 to 14.6423
3	H_2S	0.2	1.9120	0.9206 to 3.4153
		0.3	1.9185	0.9243 to 3.4264
		0.4	1.9357	0.9341 to 3.4560
4	NO_x	0.2	17.4424	7.1884 to 40.5373
		0.3	17.4306	7.1822 to 40.5153
		0.4	17.3990	7.1657 to 40.4567

Table S5: The overall impact factor and 95% confidence intervals for different CV values of the influence of impurities on corrosion in supercritical CO_2 (categorized by exposure time)

No.	Impurity	CV	Mean Effect Size	95%CI
1	O_2	0.2	0.8685	0.6251 to 1.1483
		0.3	0.8754	0.6300 to 1.1577
		0.4	0.8856	0.637 to 1.1719
2	SO_2	0.2	11.9738	9.6886 to 14.7477
		0.3	11.9411	9.7477 to 14.5821

No.	Impurity	CV	Mean Effect Size	95%CI
		0.4	11.8246	9.6257 to 14.4785
3	H_2S	0.2	1.9114	0.7325 to 3.8925
		0.3	1.9156	0.7346 to 3.9007
		0.4	1.9357	0.9341 to 3.456
4	NO_x	0.2	17.4432	6.0708 to 47.1065
		0.3	17.4348	6.0669 to 47.0888
		0.4	17.412	6.0565 to 47.0414

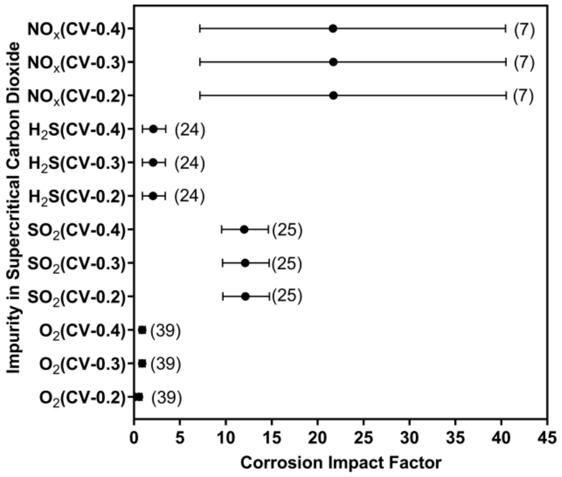

Figure S1. The influence on the general corrosion rate of carbon steels in supercritical CO_2 to O_2, SO_2, H_2S, and NO_x across subgroups of concentration at different CV

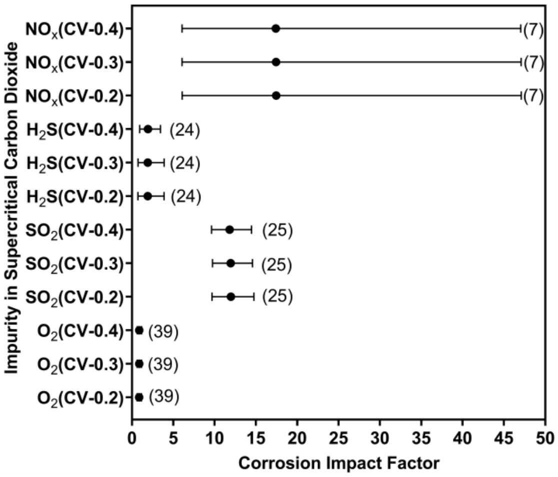

Figure S2. The influence on the general corrosion rate of carbon steels in supercritical CO_2 to O_2, SO_2, H_2S, and NO_x across subgroups of exposure time at different CV
